# Common genetic variants in *GAL, GAP43* and *NRSN1* and interaction networks confer susceptibility to Hirschsprung disease

**DOI:** 10.1111/jcmm.13612

**Published:** 2018-04-14

**Authors:** Yang Wang, Weihui Yan, Jun Wang, Ying Zhou, Jie Chen, Beilin Gu, Wei Cai

**Affiliations:** ^1^ Department of Pediatric Surgery Xinhua Hospital School of Medicine Shanghai Jiao Tong University Shanghai China; ^2^ Shanghai Key Laboratory of Pediatric Gastroenterology and Nutrition Shanghai China; ^3^ Shanghai Institute for Pediatric Research Shanghai China

**Keywords:** *GAL*, *GAP43*, Han Chinese, Hirschsprung disease, interaction networks, MassArray, *NRSN1*

## Abstract

Hirschsprung disease (HSCR) is a severe multifactorial genetic disorder. Microarray studies indicated *GAL*,*GAP43* and *NRSN1* might contribute to the altered risk in HSCR. Thus, we focused on genetic variations in *GAL*,*GAP43* and *NRSN1*, and the gene‐gene interactions involved in HSCR susceptibility. We recruited a strategy combining case‐control study and MassArray system with interaction network analysis. For *GAL*,*GAP43* and *NRSN1*, a total of 18 polymorphisms were assessed in 104 subjects with sporadic HSCR and 151 controls of Han Chinese origin. We found statistically significant differences between HSCR and control groups at 5 genetic variants. For each gene, the haplotypes combining all polymorphisms were the most significant. Based on SNPsyn, MDR and GeneMANIA analyses, we observed significant gene‐gene interactions among *GAL*,*GAP43*,*NRSN1* and our previous identified *RELN*,*GABRG2* and *PTCH1*. Our study for the first time indicates that genetic variants within *GAL*,*GAP43* and *NRSN1* and related gene‐gene interaction networks might be involved in the altered susceptibility to HSCR in the Han Chinese population, which might shed more light on HSCR pathogenesis.

## 1 INTRODUCTION

Hirschsprung disease (HSCR) is a complex genetic disorder caused by congenital defect of the enteric nervous system (ENS) which is derived from neural crest cells (NCCs). HSCR affects approximately 1/5000 live births worldwide, and the highest incidence was observed in Asian population (2.8/10 000 live births).^1^ Based on the extent of aganglionosis, the HSCR cases can be anatomically categorized into three subtypes: short segment HSCR (S‐HSCR, 80% of cases) in which the aganglionic segment does not extend beyond the upper sigmoid, long segment HSCR (L‐HSCR, 15% of cases) and total colonic aganglionosis (TCA, 5% of cases).^2^ Importantly, HSCR shows a dramatic sex bias with at least 4 times more males affected than females in S‐HSCR (male:female ≈ 1:1 in L‐HSCR) for causes that remain unclear.^3^

Genetic factors or multiple gene interactions are crucial to the development of Hirschsprung disease as HSCR is a non‐Mendelian disorder in nature with low sex‐dependent penetrance and interfamilial variation.^4^ It has been suggested that there are variations in penetrance and severity of aganglionosis between family members bearing mutations in HSCR genes.^5^ Obviously, only a single homozygous null mutation of HSCR‐related genes is insufficient to cause serious aganglionosis phenotype in HSCR.^3^ Genetic variants of at least 15 genes so far have been implicated in HSCR aetiology, including *RET* (receptor tyrosine kinase), one of the major HSCR susceptibility genes,^6^, ^7^ whereas only ~ 0.1% of the heritability in HSCR can be attributed to the mutations in these genes that account for about 50% of familial and 7%‐35% of sporadic HSCR cases,^8^ indicating more genes that might be involved in HSCR development.

On the other hand, recent genomewide association studies have revealed dozens of novel HSCR genes, which may facilitate the description of a complete landscape of genetic networks in HSCR. Taking advantage of whole exome sequencing, several genes, including *DENND3*,* FAT3* and *AGL,* were linked to HSCR pathogenesis.^9^, ^10^, ^11^ *NRG3* has recently been proved to be a new HSCR risk gene based on exome sequencing and genomewide copy number analysis,^2^, ^12^ which was further confirmed by our previous work.^13^ In addition, genomewide association studies on HSCR trios and sporadic cases have uncovered the class 3 semaphorin gene cluster and certain large‐scale chromosomal aberrations regarding HSCR aetiology.^14^, ^15^ Recent genomewide microarray analysis has reported the levels of *GAL* (galanin), *GAP43* (growth‐associated protein 43) and *NRSN1* (neurensin 1) were significantly down‐regulated in HSCR cases when compared to controls, indicating the possibility that all 3 genes might be associated with HSCR risk.^16^

**Figure 1:**
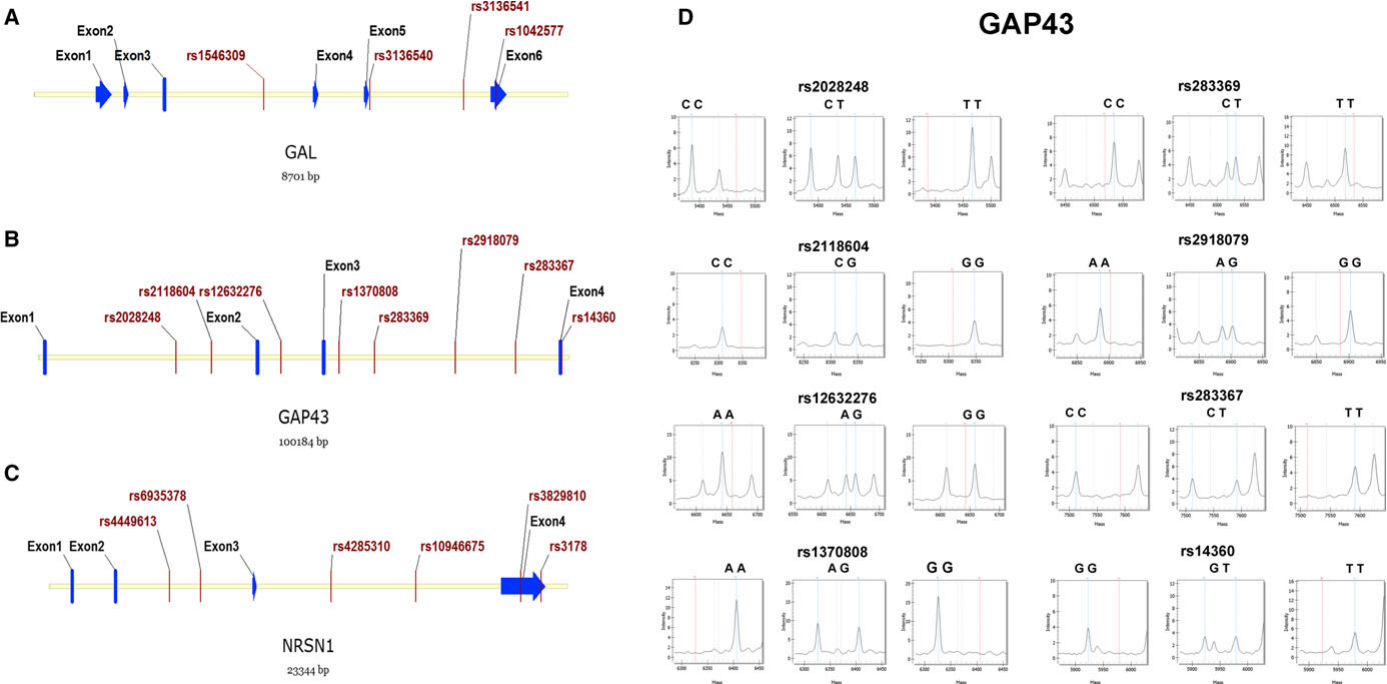
Distribution and representative mass spectra of the genetic variants in the present study. A‐C, The 18 genetic variants distributed in *GAL, GAP43* and *NRSN1*. Red lines indicate the studied SNPs; blue lines and arrows represent the exons located in the*GAL, GAP43* and *NRSN1*; D, Representative mass spectra of the 8 polymorphisms in *GAP43*. Blue dotted lines indicate the presence of the studied alleles; red dotted lines represent no allele detected; grey dotted lines denote the unrelated peaks

More importantly, joint gene‐gene effects, such as *RET* and *PHOX2B* genes, might have a crucial impact on the development of HSCR.^17^ Our previous study has proved the interactions among *GABRG2, RELN* and *PTCH1* may contribute to altered susceptibility to HSCR.^13^ Additionally, galanin‐expressing GABA neurons in the lateral hypothalamus may have important implications for treatment strategies of psychiatric disorders.^18^ In Ptch1 (+/−) mice that causes aberrant hedgehog signalling, reduced Gap43 expression leads to the Nos2‐mediated medulloblastoma development.^19^ Recently, it has been demonstrated that reelin blockade results in decreased levels of phospho‐GAP43 in the superior colliculus, suggesting the interaction of reelin signalling and phospho‐GAP43 might be involved in the development of neural circuits.^20^ With all these lines of evidence and results, we aimed to explore whether genetic variants within *GAL, GAP43* and *NRSN1* might contribute to the altered susceptibility to HSCR, and based on the 18 polymorphisms involved in this study (Figure 1A), we further assessed the interaction relationship among *GAL, GAP43, NRSN1* and our previous identified *GABRG2, RELN* and *PTCH1* genes.

## 2 MATERIALS AND METHODS

### 2.1 Subjects

The subject group involved in this study consisted of 104 cases with HSCR (84 male and 20 female) and 151 normal controls (86 male and 65 female). The mean ages of HSCR group and control group were 1.14 ± 1.83 years and 1.66 ± 1.05 years. The characteristics of the study subjects can be found in our previous study.^21^ All the participants in the study were of Han Chinese origin and were recruited from the residents who were biologically unrelated. Diagnosis of HSCR was confirmed by the histological examination of either surgical resection material or biopsy for the absence of ganglion cells. The HSCR group included 86 subjects of S‐HSCR (short segment HSCR), 15 subjects of L‐HSCR (long segment HSCR) and 3 subjects of TCA (total colonic aganglionosis). Controls were randomly enrolled from the subjects with no history of chronic constipation. The protocol of our study was reviewed and approved by the ethics committee of Xin Hua Hospital. Informed consent was obtained from parents of all participants after the procedure had been fully explained. All experiments were conducted in accordance with the tenets of the Declaration of Helsinki. DNA extraction was performed according to standard procedures with QIAamp DNA blood midi kit (Qiagen, Valencia, CA).

### 2.2 SNP selection

The tagSNP selection was conducted using the Haploview software (Version 4.2) with MAF (minor allele frequency) ≥ 0.1 and *r* ^2 ^≥ 0.8 according to the Han Chinese in Beijing (HCB) population's SNP data from the HapMap database. In regard to HCB population, we have very few choices for cSNPs (coding SNPs) and UTR SNPs because of the unavailability of allele frequency data for many polymorphisms. In our present study, we enrolled 18 tagSNPs including 4 UTR SNPs (*GAL*: rs1042577; *GAP43*: rs14360; *NRSN1*: rs3829810 and rs3178) and 14 intronic SNPs (*GAL*: rs1546309, rs3136540 and rs3136541; *GAP43*: rs2028248, rs2118604, rs12632276, rs1370808, rs283369, rs2918079 and rs283367; *NRSN1*: rs4449613, rs6935378, rs4285310 and rs10946675) (Figure 1A).

### 2.3 Genotyping and quality control

Genotyping was carried out using the MassARRAY iPLEX Gold technology (Sequenom, San Diego, CA). Briefly, PCR and iPLEX single‐base extension primers (SBE) were designed taking advantage of the Assay Design Suite of Sequenom. The whole process consisted of the PCR amplification, the shrimp alkaline phosphatase (SAP) and the primer extension reactions using iPLEX Gold assay (Sequenom) that discriminates sequence differences at the single nucleotide level. Mass signals for the different alleles were captured by MALDI‐TOF‐based system with high accuracy. Raw data from the assays were processed with Typer Version 4.0 (Sequenom).

We recruited the following criteria as a measure of acceptable genotyping: (1) 30 sample duplicates and 4 blank wells were involved in each 384‐well plate; (2) concordance rate for the duplicates ≥ 99.5%; (3) call rate for the blank wells <5% in each 384‐well plate; (4) call rate > 95% for each 384‐well plate; and (5) overall call rate by individual or by marker > 95%. The data for any marker or individual failing the criteria were excluded from further analyses.

### 2.4 SNP‐SNP interaction analysis

In this study, SNPsyn (http://snpsyn.biolab.si)^22^ was employed to interrogate the SNP‐SNP interaction networks regarding HSCR. The genotyping data of the studied genetic variants were processed with the SNPsyn software tool, using which we uncovered the SNP‐SNP interaction networks and carried out the SNP pair selection that was mainly based on information gain (I), synergy (Syn) and false discovery rate (FDR).^22^, ^23^ Moreover, the multifactor dimensionality reduction (MDR) analysis was included to explore the gene‐gene interactions. We used the MDR software version 3.0.2 to perform the MDR analysis and identified all risk factors in the best model maximizing testing accuracy and cross‐validation consistency (CVC).^24^ We further recruited the GeneMANIA database, including co‐expression, co‐localization and genetic interaction datasets, to assess the gene‐gene interaction networks and to conduct a function prediction.^25^

### 2.5 Statistical analysis

We used SHEsis (http://analysis.bio-x.cn/myAnalysis.php) to calculate Hardy‐Weinberg equilibrium, allelic and genotypic association, odds ratio (OR) and 95% confidence interval (CI) and to estimate allelic distribution and linkage disequilibrium (LD).^26^ “*D*” was included as the standardized measure for all possible pairs of SNP loci. All the *P* values in this study were two‐tailed, and the significance level was set at *P *=* *.05. Bonferroni correction was performed to correct the *P* values of genetic analysis, and Plink was enrolled to conduct the association analyses with dominant model and recessive model, and perform the adjustment for gender factor in the association analysis.^27^ Additionally, haplotype distribution was estimated using the program UNPHASED,^28^ and power calculations were conducted using the G*Power 3 program.^29^

## 3 RESULTS

In regard to the studied genetic variants, Hardy‐Weinberg equilibrium tests were conducted in HSCR group and control group, respectively. Allele and genotype frequencies of the 18 markers are listed in Tables 1, 2, 3. Genotype distributions were in Hardy‐Weinberg equilibrium for all 18 polymorphisms in either HSCR group or control group (*P *>* *.05). Power calculations were conducted in regard to all 3 genes: (1) *GAL*, the power of rs1546309, rs3136540, rs3136541 and rs1042577 was of 0.749, 0.741, 0.765 and 0.775 (OR 1.5, 95% CI); (2) *GAP43*, the power of rs2028248, rs2118604, rs12632276, rs1370808, rs283369, rs2918079, rs283367 and rs14368 was of 0.804, 0.779, 0.785, 0.8, 0.802, 0.786, 0.799 and 0.787 (OR 1.5, 95% CI); (3) *NRSN1*, the power of rs4449613, rs6935378, rs4285310, rs10946675, rs3829810 and rs3178 was of 0.804, 0.805, 0.768, 0.788, 0.791 and 0.791 (OR 1.5, 95% CI). There were significant associations between HSCR and 5 genetic polymorphisms, including 1 *GAL* SNP (rs1042577), 2 *GAP43* SNPs (rs283367 and rs14360) and 2 *NRSN1* SNPs (rs10946675 and rs3829810). We also found the significance in allele distributions of the 5 positive SNPs and in genotype distributions of the 1 *GAL* SNP and 2 *GAP43* SNPs remained after the Bonferroni correction. Moreover, all 5 positive SNPs were involved in the further analyses with dominant model (Dom) and recessive model (Rec), giving *P* values as following: (1) *GAL*_rs1042577, Dom *P *=* *.001, Rec *P *=* *.04; (2) *GAP43*_rs283367, Dom *P *=* *.064, Rec *P *=* *.001; (3) *GAP43*_rs14360, Dom *P *=* *.001, Rec*P* *=* 0.184; (4) *NRSN1*_rs10946675, Dom *P *=* *.002, Rec *P *=* *.237; and (5) *NRSN1*_rs3829810, Dom *P *=* *.013, Rec *P *=* *.049. PLINK was recruited in the adjustment for gender factor, and the findings in the 5 positive SNPs remained significant after correction. Figure 1B presents representative mass spectra of the original MassARRAY reactions in *GAP43*. Additionally, the frequencies of certain alleles and genotypes regarding the 5 positive markers were significantly higher in HSCR group compared to normal control group, such as the A allele and AA genotype of *GAL* rs1042577, the T allele and TT genotype of *GAP43* rs283367, the G allele and GG genotype of *GAP43* rs14360, the G allele and GG genotype of *NRSN1* rs10946675, and the C allele and CC genotype of *NRSN1* rs3829810.

**Table 1:** Allele and genotype distributions of *GAL* among patients with HSCR and normal controls

SNP ID	Chr (Pos)	Genotype frequency (%)			HWE check *P* value^a^	*P* value^a^	Bonferroni correction	Allele frequency (%)		*X* ^2^	*P* value^a^	Bonferroni correction	Odds ratio (95% CI)
rs1546309		CC	CT	TT				C	T				
Case	11 (68687214)	3 (3.0)	32 (32.0)	65 (65.0)	.692	.158	>0.05	38 (19.0)	162 (81.0)	3.432	.064	>0.05	1.58 (0.97‐2.58)
Control		3 (2.0)	33 (21.9)	115 (76.2)	.727			39 (12.9)	263 (87.1)				
rs3136540		CC	CT	TT				C	T				
Case	11 (68688942)	68 (67.3)	29 (28.7)	4 (4.0)	.684	.242	>0.05	165 (81.7)	37 (18.3)	2.405	.121	>0.05	0.68 (0.42‐1.11)
Control		116 (76.8)	30 (19.9)	5 (3.3)	.096			262 (86.8)	40 (13.2)				
rs3136541		CC	CT	TT				C	T				
Case	11 (68690475)	5 (4.9)	39 (37.9)	59 (57.3)	.653	.173	>0.05	49 (23.8)	157 (76.2)	3.409	.065	>0.05	1.51 (0.97‐2.35)
Control		4 (2.7)	43 (28.9)	102 (68.5)	.833			51 (17.1)	247 (82.9)				
**rs1042577**		AA	AG	GG				A	G				
Case	11 (68691002)	12 (11.9)	39 (38.6)	50 (49.5)	.313	**.003**	**0.014**	63 (31.2)	139 (68.8)	12.666	**3.75 × 10** ^**−4**^	**0.002**	2.14 (1.40‐3.27)
Control		7 (4.8)	37 (25.3)	102 (69.9)	.144			51 (17.5)	241 (82.5)				

SNP, single nucleotide polymorphism; Chr, chromosome; Pos, position; CI, confidence interval; HSCR, Hirschsprung disease; HWE, Hardy‐Weinberg equilibrium.

a Pearson's *P* value; the significance level was set at *P* = .05.

**Table 2:** Allele and genotype distributions of *GAP43* among patients with HSCR and normal controls

SNP ID	Chr (Pos)	Genotype frequency (%)			HWE check *P* value^a^	*P* value^a^	Bonferroni correction	Allele frequency (%)		*X* ^2^	*P* value^a^	Bonferroni correction	Odds ratio (95% CI)
rs2028248		CC	CT	TT				C	T				
Case	3 (115648119)	14 (14.0)	52 (52.0)	34 (34.0)	.405	.632	>0.05	80 (40.0)	120 (60.0)	0.297	.586	>0.05	0.90 (0.63‐1.30)
Control		27 (18.5)	70 (47.9)	49 (33.6)	.820			124 (42.5)	168 (57.5)				
rs2118604		CC	CG	GG				C	G				
Case	3 (115654882)	5 (4.8)	34 (32.7)	65 (62.5)	.839	.275	>0.05	44 (21.2)	164 (78.8)	2.639	.104	>0.05	0.71 (0.47‐1.08)
Control		12 (7.9)	59 (39.1)	80 (53.0)	.808			83 (27.5)	219 (72.5)				
rs12632276		AA	AG	GG				A	G				
Case	3 (115668013)	12 (11.9)	39 (38.6)	50 (49.5)	.313	.317	>0.05	63 (31.2)	139 (68.8)	2.445	.118	>0.05	1.37 (0.92‐2.04)
Control		11 (7.4)	52 (34.9)	86 (57.7)	.426			74 (24.8)	224 (75.2)				
rs1370808		AA	AG	GG				A	G				
Case	3 (115678938)	41 (40.6)	46 (45.5)	14 (13.9)	.849	.221	>0.05	128 (63.4)	74 (36.6)	1.357	.102	>0.05	1.36 (0.94‐1.96)
Control		45 (30.2)	77 (51.7)	27 (18.1)	.551			167 (56.0)	131 (44.0)				
rs283369		CC	CT	TT				C	T				
Case	3 (115685746)	33 (32.0)	51 (49.5)	19 (18.4)	.928	.939	>0.05	117 (56.8)	89 (43.2)	0.020	.887	>0.05	0.97 (0.68‐1.40)
Control		47 (31.8)	76 (51.4)	25 (16.9)	.541			170 (57.4)	126 (42.6)				
rs2918079		AA	AG	GG				A	G				
Case	3 (115700948)	51 (49.5)	42 (40.8)	10 (9.7)	.754	.697	>0.05	144 (69.9)	62 (30.1)	0.099	.753	>0.05	1.06 (0.72‐1.57)
Control		74 (50.0)	55 (37.2)	19 (12.8)	.094			203 (68.6)	93 (31.4)				
**rs283367**		CC	CT	TT				C	T				
Case	3 (115712360)	33 (32.0)	43 (41.7)	27 (26.2)	.100	**.004**	**0.035**	109 (52.9)	97 (47.1)	9.365	**.002**	**0.018**	0.57 (0.39‐0.82)
Control		65 (43.6)	68 (45.6)	16 (10.7)	.775			198 (66.4)	100 (33.6)				
**rs14360**		GG	GT	TT				G	T				
Case	3 (115721160)	10 (10.1)	51 (51.5)	38 (38.4)	.233	**.004**	**0.035**	71 (35.9)	127 (64.1)	9.708	**.002**	**0.015**	1.88 (1.26‐2.80)
Control		8 (5.6)	50 (34.7)	86 (59.7)	.836			66 (22.9)	222 (77.1)				

SNP, single nucleotide polymorphism; Chr, chromosome; Pos, position; CI, confidence interval; HSCR, Hirschsprung disease; HWE, Hardy‐Weinberg equilibrium.

a Pearson's *P* value; the significance level was set at *P* = .05.

**Table 3:** Allele and genotype distributions of *NRSN1* among patients with HSCR and normal controls

SNP ID	Chr (Pos)	Genotype frequency (%)			HWE check *P* value^a^	*P* value^a^	Bonferroni correction	Allele frequency (%)		*X* ^2^	*P* value^a^	Bonferroni correction	Odds ratio (95% CI)
rs4449613		AA	AG	GG				A	G				
Case	6 (24130593)	31 (30.7)	49 (48.5)	21 (20.8)	.840	.795	>0.05	111 (55.0)	91 (45.0)	0.067	.795	>0.05	1.05 (0.73‐1.50)
Control		40 (27.4)	77 (52.7)	29 (19.9)	.462			157 (53.8)	135 (46.2)				
rs6935378		CC	CG	GG				C	G				
Case	6 (24131987)	18 (18.9)	46 (48.4)	31 (32.6)	.898	.762	>0.05	82 (43.2)	108 (56.8)	0.525	.469	>0.05	0.87 (0.60‐1.26)
Control		31 (21.5)	72 (50.0)	41 (28.5)	.954			134 (46.5)	154 (53.5)				
rs4285310		AA	AC	CC				A	C				
Case	6 (24137874)	4 (4.0)	34 (34.0)	62 (62.0)	.805	.846	>0.05	42 (21.0)	158 (79.0)	0.015	.903	>0.05	1.03 (0.66‐1.60)
Control		4 (2.7)	52 (35.6)	90 (61.6)	.273			60 (20.5)	232 (79.5)				
**rs10946675**		AA	AG	GG				A	G				
Case	6 (24141692)	7 (7.1)	29 (29.3)	63 (63.6)	.168	**.010**	0.058	43 (21.7)	155 (78.3)	8.515	**.004**	**0.021**	0.54 (0.36‐0.82)
Control		17 (11.6)	65 (44.5)	64 (43.8)	.936			99 (33.9)	193 (66.1)				
**rs3829810**		CC	CT	TT				C	T				
Case	6 (24146444)	56 (54.4)	40 (38.8)	7 (6.8)	.968	**.022**	0.130	152 (73.8)	54 (26.2)	7.829	**.005**	**0.031**	1.74 (1.18‐2.56)
Control		57 (38.5)	69 (46.6)	22 (14.9)	.881			183 (61.8)	113 (38.2)				
rs3178		CC	CT	TT				C	T				
Case	6 (24147344)	41 (39.8)	52 (50.5)	10 (9.7)	.263	.794	>0.05	134 (65.0)	72 (35.0)	0.411	.522	>0.05	0.88 (0.61‐1.29)
Control		65 (43.6)	72 (48.3)	12 (8.1)	.194			202 (67.8)	96 (32.2)				

SNP, single nucleotide polymorphism; Chr, chromosome; Pos, position; CI, confidence interval; HSCR, Hirschsprung disease; HWE, Hardy‐Weinberg equilibrium.

a Pearson's *P* value; the significance level was set at *P* = .05.

We then performed LD and haplotype analyses of genetic variants in the 3 genes as haplotypes constructed from polymorphisms with strong LD will increase the statistical power for association with the disease. Figure S1 shows LD for each pair of SNPs in HSCR group and control group. Strong LD was observed in the following marker groups: (1) *GAL*, rs1546309‐s1042577; (2) *GAP43*, rs2118604‐rs12632276; and (3) *NRSN1*, rs4449613‐rs6935378, rs4449613‐rs3178 and rs6935378‐rs3178. We thus interrogated the haplotype distributions for these markers in the later analysis.

We selected haplotypes with strong LD for presentation (Table S1). As there were significant frequency discrepancies between HSCR and control groups, several haplotypes were observed to be strongly associated with HSCR. Additionally, haplotype analysis of these 18 polymorphisms revealed some significant global *P* values (Table S2). For each gene, the haplotypes that combined all markers were the most significant (*GAL*,* P *=* *6.78 × 10^−8^; *GAP43*,* P *=* *4.16 × 10^−12^; *NRSN1*,* P *=* *.0095). We further included G*Power 3 program in the power calculations and found our sample size had >80% power to detect a significant association (*P *<* *.05) for alleles, genotypes and haplotypes when an effect size index of 0.24 (corresponding to a “weak” gene effect) was adopted. We further compared the SNP frequency of normal controls in our present study with the SNP frequency of CHB (Han Chinese in Beijing, China) in 1000 Genomes Project Phase3 database (http://asia.ensembl.org), and no significant difference was observed between these 2 datasets (Table S3).

**Figure 2:**
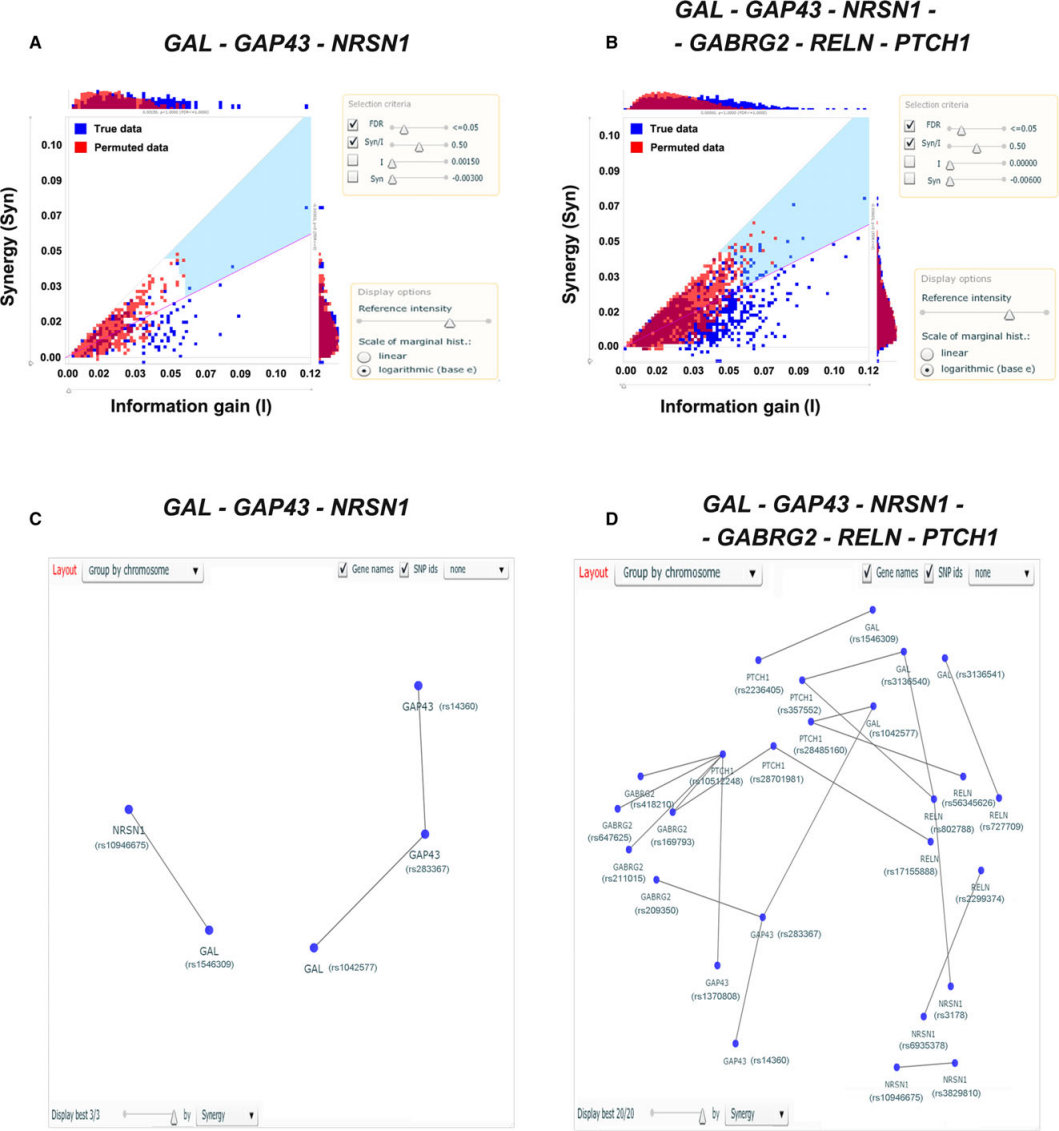
Gene‐gene interaction networks among GAL, GAP43 NRSN1 and our previous identified RELN, GABRG2 and PTCH1 gene. A and B, Distribution of SNP pair synergy (Syn) and information gain (I). The scores for SNP pairs on true data are plotted in a I vs Syn scatter plot (blue dots) with the superimposed null distribution (red dots). The SNP pairs are selected only if they meet the criteria: synergy ratio (Syn/I) ≥ 0.5 and FDR ≤ 0.05,^23^ by which the region defined is highlighted in blue. Distributions of Syn and I are plotted in histograms on the sides of the scatter plot; C and D, The interaction networks. Genes and the corresponding SNPs in the networks are connected if the SNP pairs meet the selection criteria (synergy ratio (Syn/I) ≥ 0.5 and FDR≤ 0.05); A and C, The interactions among *GAL, GAP43* and *NRSN1*; B and D, The interactions among *GAL, GAP43, NRSN1, RELN, GABRG2* and *PTCH1*

Moreover, SNPsyn software tool was enrolled to interrogate the SNP‐SNP interactions among *GAL, GAP43, NRSN1* and our previous studied *GABRG2, RELN* and *PTCH1* genes.^13^ We investigated the SNP‐SNP interaction networks based on both information gain (I) and synergy (*Syn*), and recruited only the SNP pairs with significant scores (*I, Syn*) in the network analysis (Figure 2).^22^ In our study, significant scores were found at several SNP pairs, corresponding to *GAL‐GAP43, GAL‐NRSN1, PTCH1‐GABRG2‐GAP43* group, etc. (Figure 2). The positive SNPs associated with HSCR were also involved in the SNP‐SNP interactions, such as *GAL*_rs1042577, *GAP43*_rs283367, *GAP43*_rs14360, *NRSN1*_rs10946675, *NRSN1*_rs3829810, *GABRG2*_rs209350, *GABRG2*_rs169793, *RELN*_rs802788 and *PTCH1*_rs2236405. All significant results in regard to SNPsyn analysis survived the FDR correction.

**Figure 3:**
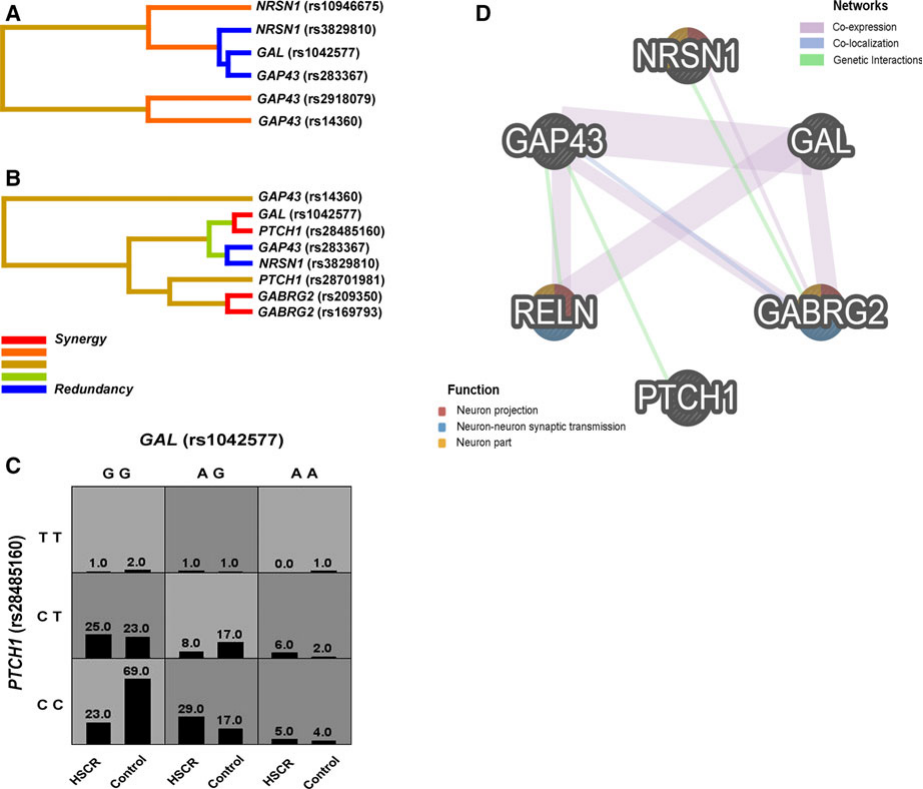
Gene‐gene interaction networks derived from MDR (multifactor dimensionality reduction) and GeneMANIA regarding HSCR risk. A and B, MDR interaction dendrogram. Shorter connections among nodes mean stronger synergistic (red and orange) or redundant (green and blue) interactions. *GAL* (rs1042577) and *PTCH1* (rs28485160) have the strongest synergistic interaction; A, The interactions between *GAL, GAP43* and *NRSN1*; B, The interactions between *GAL, GAP43, NRSN1, GABRG2, RELN* and *PTCH1*; C, Multilocus genotype combinations in the two‐factor best model are associated with the altered risks for HSCR. Each cell shows counts of HSCR cases on left and controls on right. Darker‐shaded cells show higher risk combinations when compared to lighter‐shaded cells; D, The gene network from GeneMANIA shows the relationships for *GAL, GAP43, NRSN1, GABRG2, RELN* and *PTCH1* (nodes) connected (with edges) based on the functional association networks from the databases

We further employed the MDR strategy to explore the potential gene‐gene interactions among *GAL, GAP43, NRSN1, GABRG2, RELN* and *PTCH1* corresponding to the best interaction model (Figure 3A‐C, Table 4). As for HSCR risk prediction, the best single factor model was *GAP43* (rs14360) (testing accuracy *=* 0.5813; CVC *=* 10/10), which was significantly associated with HSCR. *GAL* (rs1042577)‐*PTCH1* (rs28485160) constituted the best two‐factor model that was consistent with the results in the SNPsyn analysis. Certain genotype combinations as to *GAL* (rs1042577) and *PTCH1* (rs28485160), such as AG (rs1042577)‐CC (rs28485160), contributed to high risk in HSCR (Figure 3C). The best four‐factor model, comprising *GAP43* (rs14360), *GAP43* (rs283367), *NRSN1* (rs3829810) and *PTCH1* (rs28701981), represented the most significant one (testing accuracy *=* 0.6167; CVC *=* 7/10; OR *=* 15.27) as the accuracy and OR of the best model were increased with the rising number of factors.

**Table 4:** Gene‐gene interaction models for SNPs in HSCR risk by MDR analysis

Number of factors	Best model^a^	Training accuracy	Testing accuracy	CVC	*X* ^2^	*P* value	Odds ratio (95% CI)
1	GAP43(rs14360)	0.604	0.5813	10/10	9.592	.002	2.33 (1.36‐3.99)
2	GAL(rs1042577)‐PTCH1(rs28485160)	0.675	0.6	10/10	27.305	<.0001	4.31 (2.46‐7.57)
3	GAL(rs1042577)‐GAP43(rs283367)‐NRSN1(rs10946675)	0.7204	0.5667	10/10	43.580	<.0001	6.64 (3.70‐11.92)
4	GAP43(rs14360)‐GAP43(rs283367)‐NRSN1(rs3829810)‐PTCH1(rs28701981)	0.7947	0.6167	7/10	79.427	<.0001	15.27 (7.97‐29.25)

MDR, multifactor dimensionality reduction; CI, confidence interval; HSCR, Hirschsprung disease.

a The best model was referred to as the one with the maximum testing accuracy and maximum cross‐validation consistency (CVC).

To interrogate the functional association networks among these 6 HSCR‐related genes, we included the GeneMANIA online software in the present study using the parameters limited to co‐expression, co‐localization and genetic interactions (Figure 3D). The 6 genes interacted with each other mainly through co‐expression and genetic interactions, and only the interaction between *GAP43* and *GABRG2* was partly due to co‐localization. Moreover, gene function prediction showed *NRSN1, RELN* and *GABRG2* might contribute to neuron projection, neuron‐neuron synaptic transmission and neuron part.

## 4 DISCUSSION

HSCR is a congenital intestinal obstruction characterized by a deficit in migration of enteric neural crest cells (ENCCs), or by a defect in proliferation, differentiation or survival of ENCCs once they reach the intestinal tract.^5^ As a model of non‐Mendelian genetic disorder, HSCR can be attributed to multiple gene‐gene interactions that modulate the ability of ENCCs to populate the developing gut, and therefore, the synergistic effects of multiple hypomorphic mutations in HSCR‐related genes could affect disease penetrance and severity.^3^ However, a complete landscape of genetic networks in HSCR remains obscure. Our present study provided first evidence that genetic variants within *GAL, GAP43* and *NRSN1* might contribute to the altered susceptibility to HSCR, and the interaction networks among *GAL, GAP43, NRSN1* and our previous identified *GABRG2, RELN* and *PTCH1* genes might confer an increased risk in HSCR.

Our findings suggested a significant association of *GAL* (rs1042577) with the altered susceptibility to HSCR. As rs1042577 is located in the untranslated region, this genetic variant may exert an impact on the regulatory mechanisms of gene expression.^30^ Moreover, we observed that the G allele and GG genotype of rs1042577 were less frequent in HSCR group compared to normal control group, which indicated that the G allele and GG genotype might be involved in a protective effect against HSCR, and yet the A allele and AA genotype of rs1042577 were more common in HSCR cases than in controls, implying that all might be the risk factors for HSCR. Galanin encoded by *GAL* is a neuroendocrine peptide, which is widely expressed in the central and peripheral nervous systems and also the gastrointestinal tract.^31^ Additionally, galanin modulates transmitter release from myenteric neurons via inhibition of voltage‐dependent calcium channels mediated by G‐protein‐coupled receptors.^32^ A recent genomewide study^16^ suggested *GAL* as a candidate for HSCR due to the reduced level of *GAL* expression in HSCR group compared with control group, and our results further supported this opinion.

We further interrogated the association between *GAP43* and HSCR, and found that 2 genetic markers (rs283367 and rs14360) within *GAP43* gene presented a strong association with the HSCR risk. Saeed et al^16^ have pointed out that *GAP43* was significantly down‐regulated in the diseased segment of HSCR cases compared to controls. The protein encoded by *GAP43* is expressed at high levels in neuronal growth cones during development and axonal regeneration, suggesting its presynaptic localization in developing neurons.^33^ It has been suggested that GAP43 is a crucial component of an effective regenerative response in the nervous system, and the interaction of GAP43 and MASH1/Ascl1a (the basic helix‐loop‐helix transcription factor) promote functional axon regeneration in the adult central nervous system (CNS).^34^ Based on our data, the T allele and TT genotype of rs283367 and the G allele and GG genotype of rs14360 might be the risk factors involved in HSCR pathogenesis, whereas the C allele and CC genotype of rs283367 and the T allele and TT genotype of rs14360 might be the protective factors against HSCR. As a UTR SNP, rs14360 might play a crucial role in modulating the level of *GAP43* expression. Although it is located in the intronic region of *GAP43*, rs283367 might still have an effect on the gene expression.

On the other hand, we tried to assess the relationship between *NRSN1* gene and HSCR risk. As rs10946675 and rs3829810, the 2 positive SNPs found within *NRSN1,* were located in the intronic and untranslated regions, respectively, these polymorphisms might be involved in the regulatory mechanisms of gene expression. Specifically, we noticed the G allele and GG genotype of rs10946675 and the C allele and CC genotype of rs3829810 were more frequent in HSCR group than in control group, indicating that all may contribute to the altered risk of HSCR. Neurensin 1 (NRSN1) is a neuron‐specific protein comprising one microtubule‐binding domain and several membrane domains, and it is particularly abundant in neuronal processes, such as the process of neurite extension.^35^ NRSN1, as a key regulator, may function in neuronal organelle transport and in the conduction of nerve signals, therefore contributing to axonal regeneration and development.^36^ In addition, the expression of *NRSN1* has been proved to be down‐regulated in HSCR cases compared with normal controls,^16^ further supporting *NRSN1* gene as a potential susceptibility gene to HSCR.

In the present study, the significance regarding haplotypes might contribute to the altered risk of HSCR as well (Table S1), as under certain conditions haplotype analysis may increase the power to detect disease loci compared with the single SNP analysis.^37^ As for each of the 3 genes, the most significant haplotype involved all genetic variants in the corresponding gene (Table S1). Interestingly, certain significant haplotypes might be the protective factors in HSCR, such as *GAL*_T‐C‐T‐G (rs1546309‐rs3136540‐rs3136541‐rs1042577, *P* *=* 1.62 × 10^−5^, OR *=* 0.43, 95% CI 0.29‐0.64).

As the interactions among GAL, GABA signalling, GAP43, PTCH1 and reelin might play a crucial role in the neural functions and related disease processes,^18^, ^19^, ^20^ we utilized the SNPsyn platform to further interrogate the SNP‐SNP interaction networks among *GAL, GAP43, NRSN1* and our previous identified *GABRG2, RELN* and *PTCH1* genes. On the other hand, synergistic combinations carry more information compared to the sum of information contained in individual SNPs and specifically may carry information in regard to the phenotypes.^38^ In the present study, we found significant interaction networks in the *GAL‐GAP43‐NRSN1* and *GAL‐GAP43‐NRSN1‐GABRG2‐RELN‐PTCH1* group, respectively (Figure 2). All the 5 positive genetic variants observed within *GAL, GAP43* and *NRSN1* were involved in the interaction networks (Figure 2C,D). Moreover, we noticed that certain SNP pairs involved in the networks were located within the same gene, such as rs283367‐rs14360 (*GAP43*), indicating that the *cis*‐regulation effect might facilitate this kind of SNP‐SNP interaction.^39^

We further recruited multifactor dimensionality reduction (MDR) method to evaluate the gene‐gene interactions on the risk of HSCR using the data in regard to the 6 HSCR‐associated genes. MDR was a nonparametric approach that does not require specification of a genetic model to detect gene‐gene interactions without main gene effects.^40^ Taking advantage of the MDR analysis, we assessed the best interaction model with the maximum testing accuracy and maximum CVC between all the genes involved in our study. Of note, all the positive SNPs within *GAL, GAP43* and *NRSN1* were included in the best models obtained from the MDR analysis (Table 4). Specifically, the best two‐factor model, *GAL* (rs1042577)‐*PTCH1* (rs28485160), was also identified in the SNPsyn analysis (Figure 2). Compared with other models, the best four‐factor model, *GAP43* (rs14360)‐*GAP43* (rs283367)‐*NRSN1* (rs3829810)‐*PTCH1* (rs28701981), presented the most significant OR, raising the possibility that a multifactor model was more likely to facilitate the increased risk to HSCR.

By utilizing the GeneMANIA approach, we further explore the functional networks between these 6 genes involved in HSCR risk (Figure 3D). These 6 genes functionally connected to each other via co‐expression, co‐localization and genetic interactions, and in particular *NRSN1, RELN* and *GABRG2* were predicted to be involved in the processes of neuron projection, neuron‐neuron synaptic transmission and neuron part, further supporting all might contribute to the development of HSCR as HSCR is essentially caused by the defects in the enteric nervous system.

To sum up, our findings firstly demonstrated that genetic variants within *GAL, GAP43* and *NRSN1* might contribute to the altered susceptibility to HSCR in the Han Chinese population. The interaction networks among *GAL, GAP43, NRSN1* and our previous identified *GABRG2, RELN* and *PTCH1* genes might be involved in the risk of HSCR, and specifically, the interactions between *GAP43, NRSN1* and *PTCH1* might confer the increased risk to HSCR. Our present study points to the need for more independent replication studies with more markers and larger sample size in other ethnic groups. Finally, describing the complete landscape of genetic networks in the pathogenesis of HSCR will definitely depend on technological and conceptual advances.

## CONFLICT OF INTEREST

The authors confirm that there are no conflict of interests.
