# Supplementary figures and images for: Common genetic variants in GAL, GAP43 and NRSN1 and interaction networks confer susceptibility to Hirschsprung disease
Source: J Cell Mol Med. 2018 Apr 14;22(7):3377–87. doi: 10.1111/jcmm.13612 (PMC6010875; doi:10.1111/jcmm.13612)

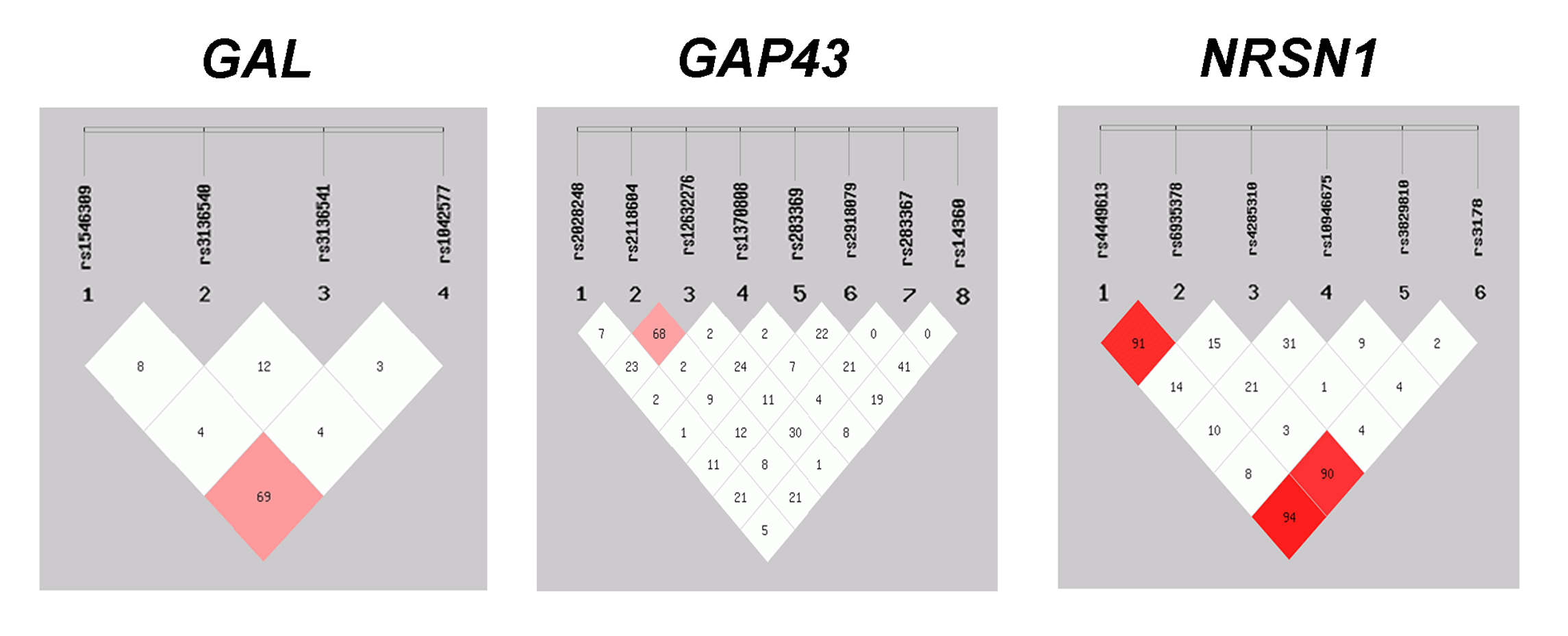

Supplement: Supplementary file 1 [file JCMM-22-3377-s001.tif]
