# Supplementary material for: Common genetic variants in GAL, GAP43 and NRSN1 and interaction networks confer susceptibility to Hirschsprung disease
Source: J Cell Mol Med. 2018 Apr 14;22(7):3377–87. doi: 10.1111/jcmm.13612 (PMC6010875; doi:10.1111/jcmm.13612)
Supplement: Supplementary file 2 [file JCMM-22-3377-s002.docx]

**Figure legends**

**Fig.S1** Linkage disequilibrium (LD) among the genetic variants within *GAL*, *GAP43* or *NRSN1*. D' value between the studied SNP pairs is denoted by the matrices. SNP = single nucleotide polymorphisms.

| **Table S1**Estimated haplotype frequencies and association significance | | | | | | | | | | | | |  |
| --- | --- | --- | --- | --- | --- | --- | --- | --- | --- | --- | --- | --- | --- |
| Gene ID | Haplotype* | | | | | | | | Haplotype frequency(%) | | X^2^ | *p* value | Odds Ratio (95%CI) |
| *GAL* | rs1546309 | rs3136540 | rs3136541 | rs1042577 |  |  |  |  | HSCR | Control |  |  |  |
|  | T |  |  | A |  |  |  |  | 56.68(28.9) | 50.69(17.4) | 9.749 | **0.002** | 1.98(1.29-3.06) |
|  | T |  |  | G |  |  |  |  | 101.32(51.7) | 204.31(70.0) | 15.194 | **9.79 x 10^-5^** | 0.47(0.33-0.69) |
|  | T | C | T | A |  |  |  |  | 41.53(21.2) | 33.73(11.6) | 8.550 | **0.003** | 2.09(1.27-3.44) |
|  | T | C | T | G |  |  |  |  | 65.29(33.3) | 154.16(52.8) | 18.616 | **1.62 x 10^-5^** | 0.43(0.29-0.64) |
| *GAP43* | rs2028248 | rs2118604 | rs12632276 | rs1370808 | rs283369 | rs2918079 | rs283367 | rs14360 |  |  |  |  |  |
|  |  | C | A |  |  |  |  |  | 43.00(21.3) | 54.66(18.3) | 0.665 | 0.415 | 1.20(0.77-1.88) |
|  | C | G | G | A | T | A | C | T | 1.40(0.7) | 10.10(3.6) | 4.825 | **0.028** | 0.17(0.03-1.01) |
|  | T | G | G | G | T | A | C | T | 1.65(0.9) | 11.04(3.9) | 5.070 | **0.024** | 0.18(0.03-0.95) |
| *NRSN1* | rs4449613 | rs6935378 | rs4285310 | rs10946675 | rs3829810 | rs3178 |  |  |  |  |  |  |  |
|  | A | G |  |  |  | T |  |  | 65.00(34.6) | 83.8(29.5) | 1.217 | 0.270 | 1.25(0.84-1.86) |
|  | A | G | A | G | T | C |  |  | 0.87(0.5) | 11.28(4.0) | 5.096 | **0.024** | 0.10(0.02-0.65) |
|  | G | C | C | A | C | C |  |  | 11.89(6.5) | 30.30(10.7) | 2.921 | 0.087 | 0.55(0.27-1.10) |
|  | G | C | C | A | T | C |  |  | 2.73(1.5) | 18.13(6.4) | 6.811 | **0.009** | 0.21(0.06-0.76) |
| *Haplotypes were omitted from analysis if the estimated haplotype probabilities were less than 3%, CI = confidence interval, HSCR = Hirschsprung disease. | | | | | | | | | | | | | |

| **Table S2**Global p values of estimated haplotypes | | |  |  |  |  |
| --- | --- | --- | --- | --- | --- | --- |
| Gene ID | Haplotype | | | |  | Global *p* value* |
| *GAL* | rs1546309-rs1042577 | | | | | **0.0004** |
|  | rs1546309-rs3136540-rs3136541-rs1042577 | | | | | **6.78 x 10^-8^** |
| *GAP43* | rs2118604-rs12632276 | | | | | **7.51 x 10^-5^** |
|  | rs2028248-rs2118604-rs12632276-rs1370808-rs283369-rs2918079-rs283367-rs14360 | | | | | **4.16 x 10^-12^** |
| *NRSN1* | rs4449613-rs6935378 | | | | | 0.5258 |
|  | rs4449613-rs3178 | | | | | 0.7892 |
|  | rs6935378-rs3178 |  |  |  |  | 0.6560 |
|  | rs4449613-rs6935378-rs3178 | |  |  |  | 0.5290 |
|  | rs4449613-rs6935378-rs4285310-rs10946675-rs3829810-rs3178 | | | |  | **0.0095** |
| *Pearson's *p* value, statistical significance set at *p*<0.05, SNP = single nucleotide polymorphism. | | | | | | |

| **Table S3** SNP frequency comparison between controls in the present study and 1000 Genomes Project Phase3 | | | | | | |
| --- | --- | --- | --- | --- | --- | --- |
| Gene ID | SNP ID | Genotype frequency (%) | | | Allele frequency(%) | |
| *GAL* | rs1546309 | CC | CT | TT | C | T |
|  | 1000 Genomes | 1.9 | 24.3 | 73.8 | 14.1 | 85.9 |
|  | Control | 2.0 | 21.9 | 76.2 | 12.9 | 87.1 |
|  | rs3136540 | CC | CT | TT | C | T |
|  | 1000 Genomes | 73.8 | 24.3 | 1.9 | 85.9 | 14.1 |
|  | Control | 76.8 | 19.9 | 3.3 | 86.8 | 13.2 |
|  | rs3136541 | CC | CT | TT | C | T |
|  | 1000 Genomes | 2.9 | 33.0 | 64.1 | 19.4 | 80.6 |
|  | Control | 2.7 | 28.9 | 68.5 | 17.1 | 82.9 |
|  | rs1042577 | AA | AG | GG | A | G |
|  | 1000 Genomes | 2.9 | 33.0 | 64.1 | 19.4 | 80.6 |
|  | Control | 4.8 | 25.3 | 69.9 | 17.5 | 82.5 |
| *GAP43* | rs2028248 | CC | CT | TT | C | T |
|  | 1000 Genomes | 26.2 | 49.5 | 24.3 | 51.0 | 49.0 |
|  | Control | 18.5 | 47.9 | 33.6 | 42.5 | 57.5 |
|  | rs2118604 | CC | CG | GG | C | G |
|  | 1000 Genomes | 7.8 | 35.9 | 56.3 | 25.7 | 74.3 |
|  | Control | 7.9 | 39.1 | 53.0 | 27.5 | 72.5 |
|  | rs12632276 | AA | AG | GG | A | G |
|  | 1000 Genomes | 8.7 | 42.7 | 48.5 | 30.1 | 69.9 |
|  | Control | 7.4 | 34.9 | 57.7 | 24.8 | 75.2 |
|  | rs1370808 | AA | AG | GG | A | G |
|  | 1000 Genomes | 31.1 | 49.5 | 19.4 | 55.8 | 44.2 |
|  | Control | 30.2 | 51.7 | 18.1 | 56.0 | 44.0 |
|  | rs283369 | CC | CT | TT | C | T |
|  | 1000 Genomes | 31.1 | 49.5 | 19.4 | 55.8 | 44.2 |
|  | Control | 31.8 | 51.4 | 16.9 | 57.4 | 42.6 |
|  | rs2918079 | AA | AG | GG | A | G |
|  | 1000 Genomes | 51.5 | 39.8 | 8.7 | 71.4 | 28.6 |
|  | Control | 50.0 | 37.2 | 12.8 | 68.6 | 31.4 |
|  | rs283367 | CC | CT | TT | C | T |
|  | 1000 Genomes | 51.5 | 39.8 | 8.7 | 71.4 | 28.6 |
|  | Control | 43.6 | 45.6 | 10.7 | 66.4 | 33.6 |
|  | rs14360 | GG | GT | TT | G | T |
|  | 1000 Genomes | 4.9 | 35.0 | 60.2 | 22.3 | 77.7 |
|  | Control | 5.6 | 34.7 | 59.7 | 22.9 | 77.1 |
| *NRSN1* | rs4449613 | AA | AG | GG | A | G |
|  | 1000 Genomes | 32.0 | 48.5 | 19.4 | 56.3 | 43.7 |
|  | Control | 27.4 | 52.7 | 19.9 | 53.8 | 46.2 |
|  | rs6935378 | CC | CG | GG | C | G |
|  | 1000 Genomes | 18.4 | 48.5 | 33.0 | 42.7 | 57.3 |
|  | Control | 21.5 | 50.0 | 28.5 | 46.5 | 53.5 |
|  | rs4285310 | AA | AC | CC | A | C |
|  | 1000 Genomes | 5.8 | 32.0 | 62.1 | 21.8 | 78.2 |
|  | Control | 2.7 | 35.6 | 61.6 | 20.5 | 79.5 |
|  | rs10946675 | AA | AG | GG | A | G |
|  | 1000 Genomes | 11.7 | 44.7 | 43.7 | 34.0 | 66.0 |
|  | Control | 11.6 | 44.5 | 43.8 | 33.9 | 66.1 |
|  | rs3829810 | CC | CT | TT | C | T |
|  | 1000 Genomes | 48.5 | 45.6 | 5.8 | 71.4 | 28.6 |
|  | Control | 38.5 | 46.6 | 14.9 | 61.8 | 38.2 |
|  | rs3178 | CC | CT | TT | C | T |
|  | 1000 Genomes | 43.7 | 44.7 | 11.7 | 66.0 | 34.0 |
|  | Control | 43.6 | 48.3 | 8.1 | 67.8 | 32.2 |
| SNP = single nucleotide polymorphism, HSCR = Hirschsprung disease, 1000 Genomes = CHB (Han Chinese in Beijing, China) in 1000 Genomes Project Phase3 database. | | | | | | |
